# Preparation and Sound Absorption Properties of a Barium Titanate/Nitrile Butadiene Rubber–Polyurethane Foam Composite with Multilayered Structure

**DOI:** 10.3390/ma11040474

**Published:** 2018-03-22

**Authors:** Xueliang Jiang, Zhen Yang, Zhijie Wang, Fuqing Zhang, Feng You, Chu Yao

**Affiliations:** College of Materials Science and Engineering, Wuhan Institute of Technology, Wuhan 430074, China; jiangxl@wit.edu.cn (X.J.); tabjpz@163.com (Z.Y.); wzj0038@126.com (Z.W.); zhangfq@wit.edu.cn (F.Z.); youfeng.mse@wit.edu.cn (F.Y.)

**Keywords:** polymeric composites, multilayered structure, BaTiO_3_/NBR, PU foam, low-frequency sound absorption

## Abstract

Barium titanate/nitrile butadiene rubber (BT/NBR) and polyurethane (PU) foam were combined to prepare a sound-absorbing material with an alternating multilayered structure. The effects of the cell size of PU foam and the alternating unit number on the sound absorption property of the material were investigated. The results show that the sound absorption efficiency at a low frequency increased when decreasing the cell size of PU foam layer. With the increasing of the alternating unit number, the material shows the sound absorption effect in a wider bandwidth of frequency. The BT/NBR-PU foam composites with alternating multilayered structure have an excellent sound absorption property at low frequency due to the organic combination of airflow resistivity, resonance absorption, and interface dissipation.

## 1. Introduction

In recent years, scholars have carried out many studies about various sound-absorbing materials, and sound-absorbing materials with a polymer matrix received much attention due to their damping effect and low density [1,2,3]. Meanwhile, it was found that the sound absorption property of materials with a polymer matrix was dependent on the polymer properties, filler, and structure. The inefficiency of low-frequency sound absorption has become an important factor restricting the development of sound-absorbing material. Compared with high-frequency sound waves, the low-frequency sound waves are hard to absorb due to the slow attenuation in the air and the interference of wind or obstacles. Furthermore, low-frequency noise is no less harmful to human body than high-frequency noise and will result in neurasthenia, insomnia, headache, and other neuroses [4,5].

Various kinds of rubber and plastic were tested as the matrix, and several fillers were proposed to improve the sound absorption property, such as ramie fiber [6], sugar cane bagasse [7], piezoceramics [8], and so on. Moreover, the structural design of materials is another strategy to increase the sound absorption property. Many structures were investigated such as periodic [9,10], helical [11], porous [12,13], multilayered [14], and so on. Phonon crystal, a kind of material with a periodic structure, is deemed an ideal material to shield noise. Phonon crystal could restrain the propagation of elastic waves in a specific frequency range, which is known as the band gap. This is practical for shielding sound waves by using phonon crystal from local resonance theory [15] about the formation of the band gap, but most relevant studies remain at the theoretical phase, using computer simulations only. In summary, it is difficult to increase the low-frequency sound absorption efficiency of materials by altering the formula (matrix and filler) or simplex structure. Therefore, multiple mechanisms and structures should be combined in the design.

A lot of work has been done on the preparation of inorganic nanoparticles and the combination of polymers to obtain functional polymer composites [16,17,18,19,20,21,22,23,24,25,26,27,28,29]. In this study, a new type of material with a multilayered structure was prepared to absorb sound. Polyurethane (PU) foam was used as a layer, and barium titanate/nitrile butadiene rubber (BT/NBR) composite was used as another layer. The layered composite consisted of alternating layers of PU foam and BT/NBR composite. The effects of the cell size of PU foam and alternating unit number on the sound absorption of the material were studied.

## 2. Materials and Methods

Nitrile rubber (NBR, N41) was produced by Lanzhou Petrochemical (Lanzhou, China). Polyurethane (PU) foam was produced by Xingtaili New Energy Materials Factory (Kunshan, China). Conductive carbon black was provided by Tianjin Ebory Chemical Co. Ltd. (Tianjin, China). BaTiO_3_ powder (BT) was prepared by the authors. Other ingredients were bought locally.

**Figure 1:**
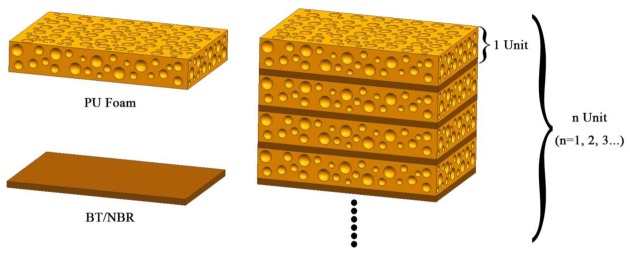
Schematic of BT/NBR-PU foam composite.

**Figure 2:**
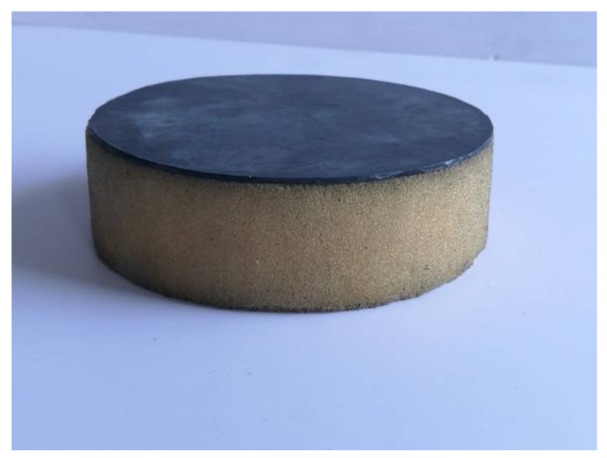
Actual shape of BT/NBR-PU foam composite.

First, 100 phr NBR, 5 phr ZnO, 1.5 phr Stearic acid, 2 phr tetramethylthiuram disulfide (Accelerator TMTD), 1 phr N-Cyclohexyl-2-benzothiazolylsulfenamide (Accelerator CZ), 1 phr Antiager 4010, 12 phr Conductive carbon black, 99 phr BT and 1.5 phr Sulfur were mixed in a two-roll mill (SK160B, Shanghai Tuolin Rubber Machinery Factory, Shanghai, China). The BT/NBR composites were vulcanized with a plate vulcanization machine (Huzhou Oriental Machinery Co. Ltd., Huzhou, China) at 10 MPa and 160 °C for 15 min. Then the BT/NBR composites and PU foam composites were combined with an adhesive (as in Figure 1). The actual shape of the sample is shown in Figure 2.

The morphology of polyurethane foam was observed with an optical microscope. Sound absorption was tested by an AWA6128A-type standing wave tube (Beijing Century JT Technology Development Co. Ltd., Beijing, China). 

## 3. Results and Discussion

**Figure 3:**
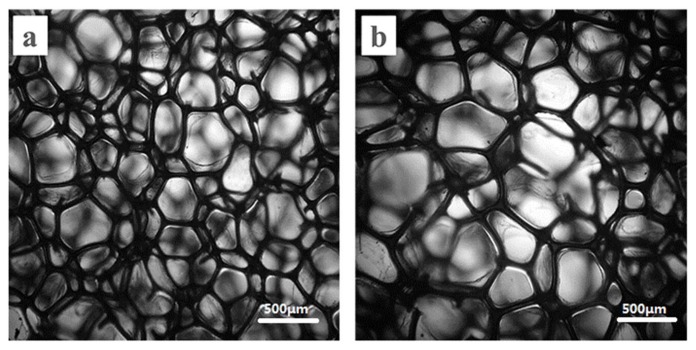
Optical microscope image ((**a**) PUSF; (**b**) PUBF).

**Figure 4:**
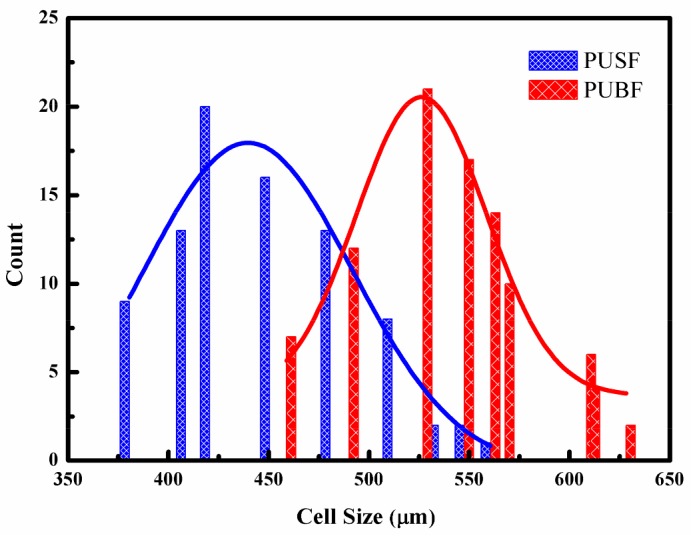
Cell distributions of PU foam composites.

In order to investigate the effect of the cell size of a PU foam layer on the sound absorption property of the materials, two kinds of PU foam composites were used in the sound absorption composites: a PU foam composite with big bubble holes (PUBF composite) and a PU foam composite with small bubble holes (PUSF composite). The bubble morphology and the size distribution of the bubble holes are shown in Figure 3 and Figure 4. The distributions of the bubble holes are uniform, and the cells of PUSF are smaller than the cells of PUBF. The bubble holes’ mean diameter in the PUSF composites is 445.4 μm and in the PUBF composites it is 541.4 μm.

**Figure 5:**
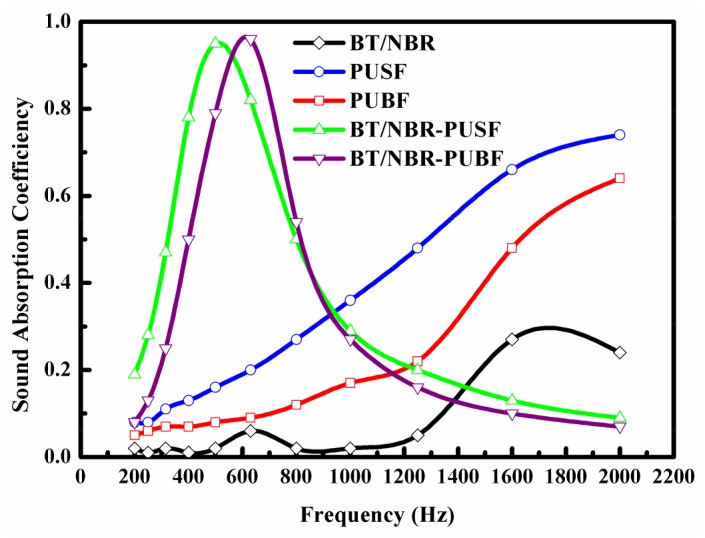
Sound absorption efficiencies of BT/NBR, PUSF, PUBF, BT/NBR-PUSF (*n* = 1), and BT/NBR-PUBF (*n* = 1) composites.

The sound absorption efficiencies of BT/NBR, PUSF, PUBF, BT/NBR-PUSF (*n* = 1), and BT/NBR-PUBF (*n* = 1) composites are shown in Figure 5. The thickness of the BT/NBR layer is 2 mm and the thickness of the PU foam layer is 25 mm. The sound absorption efficiency of the BT/NBR composite is low, especially at a low frequency. The sound absorption efficiencies at low frequency of the PU foam composites are also low, whether the cells are small or big. However, both the BT/NBR-PUSF composite and the BT/NBR-PUBF composite show excellent sound absorption at low frequency. On the one hand, good absorption at low frequency benefits from the resonance of the mass per unit area of the nitrile rubber with the combined stiffness of the frame of the polyurethane foam and the air in the holes of the polyurethane foam. On the other hand, the PU foam layer with high airflow resistivity has good sound absorption at medium and high frequency. However, the PU foam layer could not effectively absorb the sound wave at low frequency alone, as the sound wave at low frequency has high penetrability. The existence of a BT/NBR layer with high density could prevent the penetration of the sound wave at low frequency and improve the airflow resistivity [30,31,32]. The BT/NBR layer could also reflect part of the sound wave, which leads to a longer propagation path of the sound wave in the PU foam layer. Therefore, the BT/NBR-PU foam material with a multilayered structure shows good absorption at low frequency. Furthermore, the sound absorption peak of the material moves to low frequency when decreasing the cell size of PU foam layer. The energy causing the cell resonance decreased with a decrease in the cell size. Therefore, a low-frequency sound wave with low energy could be absorbed effectively by a material with small cells of a PU foam layer.

**Figure 6:**
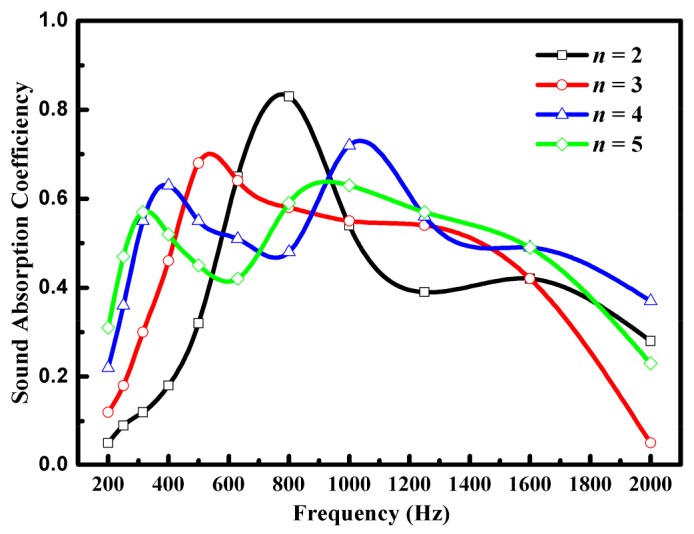
Effect of layer number on sound absorption efficiency of the BT/NBR-PUSF composites.

The effects of alternating unit number on the sound absorption efficiency of the BT/NBR-PUSF composites are shown in Figure 6. Considering that the aggregate thickness of the material should not be too large, the thickness of PU foam layer was decreased from 25 mm to 5 mm. With an increase in the alternating unit number, the material shows the sound absorption effect in a wider bandwidth of frequency. The alternating multilayered structure provides more interfaces when increasing of the alternating unit number, so more reflection and friction loss happened on these interfaces [14]. Moreover, the airflow resistivity of the composites would improve when increasing the alternating unit number, and the propagation path of the sound wave would also become longer. Therefore, the resonance absorption and interface dissipation of the sound wave became more obvious.

It is difficult to increase the low-frequency sound absorption efficiency of materials by simply altering the fillers such as ramie fiber [6], sugar cane bagasse [7], piezoceramics [8], and so on. In works by previous researchers, the sound-absorbing effects at a frequency above 1000 Hz could be good, but those at a frequency below 1000 Hz are weak. The low-frequency sound absorption efficiencies of materials by structure design were better but not good enough. At a frequency below 1000 Hz, the average sound absorption coefficient of materials with periodic structure [9,10], helical structure [11], or porous structure [12,13] was below 0.3, and the sound absorption coefficient was below 0.6 at a specific frequency. Furthermore, foam/film poly (ethylene-co-octene) composites with a multilayered structure had been reported, but the low-frequency sound absorption efficiency of the composites is poor [14]. However, at a frequency below 1000 Hz, the average sound absorption coefficient of the BT/NBR-PUSF composites with a multilayered structure could be above 0.5, and the sound absorption coefficient could be above 0.95 at a specific frequency. Therefore, the interaction of elasticity matrix, suitable filler, and multilayered structure leads to the organic combination of airflow resistivity, resonance absorption, and interface dissipation, which could effectively improve the sound absorption of the material.

## 4. Conclusions

A BT/NBR-PU foam material with a multilayered structure was successfully prepared and shows excellent sound absorption at low frequency. When decreasing the cell size of the PU foam layer, the sound absorption peak of the material moves to low frequency. When increasing the alternating unit number, the material shows sound absorption in a wider bandwidth of frequency. The alternating multilayered structure provides more interfaces when increasing the alternating unit number, which leads to more reflection and friction loss on interfaces, higher airflow resistivity, and a longer propagation path of sound. The BT/NBR-PUSF (*n* = 4, 5) composites had the best sound absorption efficiency, and the BT/NBR-PUSF (*n* = 1) composites had the best sound absorption efficiency at a specific frequency. Furthermore, the organic combination of airflow resistivity, resonance absorption, and interface dissipation could effectively improve the sound absorption of the material.

## References

[1] Yang M., Sheng P. (2017). Sound absorption structures: From porous media to acoustic metamaterials. Annu. Rev. Mater. Res..

[2] Ru J.M., Kong B., Liu Y.G., Wang X.L., Fan T.X., Zhang D. (2015). Microstructure and sound absorption of porous copper prepared by resin curing and foaming method. Mater. Lett..

[3] Liu P.S., Qing H.B., Hou H.L. (2015). Primary investigation on sound absorption performance of highly porous titanium foams. Mater. Des..

[4] Richoux O., Ayrault C., Pelat A., Félix S., Lihoreau B. (2009). Effect of the open roof on low frequency acoustic propagation in street canyons. Appl. Acoust..

[5] Sousa A.N., Gibbs B.M. (2011). Low frequency impact sound transmission in dwellings through homogeneous concrete floors and floating floors. Appl. Acoust..

[6] Chen D., Li J., Ren J. (2010). Study on sound absorption property of ramie fiber reinforced poly(l-lactic acid) composites: Morphology and properties. Compos. Part A.

[7] Doost-Hoseini K., Taghiyari H.R., Elyasi A. (2014). Correlation between sound absorption coefficients with physical and mechanical properties of insulation boards made from sugar cane bagasse. Compos. Part B.

[8] Wu C.M., Chou M.H. (2016). Polymorphism, piezoelectricity and sound absorption of electrospun PVDF membranes with and without carbon nanotubes. Compos. Sci. Technol..

[9] Yang X.H., Ren S.W., Wang W.B., Liu X., Xin F.X., Lu T.J. (2015). A simplistic unit cell model for sound absorption of cellular foams with fully/semi-open cells. Compos. Sci. Technol..

[10] Maldovan M. (2013). Sound and heat revolutions in phononics. Nature.

[11] Babaee S., Viard N., Wang P., Fang N.X., Bertoldi K. (2016). Harnessing deformation to switch on and off the propagation of sound. Adv. Mater..

[12] Xue B., Li R., Deng J.G., Zhang J.H. (2016). Sound absorption properties of microporous poly(vinyl formal) foams prepared by a two-step acetalization method. Ind. Eng. Chem. Res..

[13] Zhai W., Yu X., Song X., Ang L.Y.L., Cui F., Lee H.P., Li T. (2018). Microstructure-based experimental and numerical investigations on the sound absorption property of open-cell metallic foams manufactured by a template replication technique. Mater. Des..

[14] Zhao T.B., Yang M.T., Wu H., Guo S.Y., Sun X.J. (2015). Preparation of a new foam/film structure poly (ethylene-co-octene) foam materials and its sound absorption properties. Mater. Lett..

[15] Liu Z.Y., Zhang X.X., Mao Y.W., Zhu Y.Y., Yang Z.Y., Chan C.T., Sheng P. (2000). Locally resonant sonic materials. Science.

[16] Lu T., Pan H., Ma J., Li Y., Bokhari S.W., Jiang X., Zhu S., Zhang D. (2017). Cellulose nanocrystals/polyacrylamide composites of high sensitivity and cycling performance to gauge humidity. ACS Appl. Mater. Interfaces.

[17] Jiang X., Li C., Liu S., Zhang F., You F., Yao C. (2017). The synthesis and characterization of ytterbium-doped TiO_2_ hollow spheres with enhanced visible-light photocatalytic activity. Rsc. Adv..

[18] Chen R., Jiang X., You F., Yao C. (2016). Optimizing the morphology, mechanical and crystal properties of in-situ polypropylene/polystyrene blends by reactive extrusion. Fibers Polym..

[19] Jiang X., Xu X., Geng T., You F., Wang W., Yao C. (2016). Fabrication of a novel water swellable styrene-butadiene rubber through the in-situ polymerization of lithium acrylat. Polym. Korea.

[20] Jiang X., Zhang J., Yu L., You F. (2016). Yttria hollow nano-flowers synthesized by hydrothermal method and their adsorption capacity. Chin. J. Inorg. Chem..

[21] Jiang X., Yu L., Yao C., You F., Zhang J. (2016). Facile fabrication and characterization of ytterbium oxide hollow spheres using carbon spheres as template. Nano.

[22] Jiang X., Yu L., Yao C., Zhang F., Zhang J., Li C. (2016). Synthesis and characterization of Gd_2_O_3_ hollow microspheres using a template-directed method. Materials.

[23] Jiang X., Zhang J., Yu L., Chen R. (2016). Synthesis of mono-dispersed ceria hollow nanospheres by a hydrothermal method. Micro Nano Lett..

[24] Jiang X., Wang W., Guan J., Yang H., Sun G., Ren J. (2015). Preparation of CeO_2_ hollow nanospheres by PMMA template and their low frequency damping capacity. Rare Metal Mater. Eng..

[25] Jiang X., Du Y., Wang W., Yang H., Ren J. (2014). Preparation of Y_2_O_3_ hollow nanospheres by homogeneous precipitation templates method. Rare Metal Mater. Eng..

[26] Jiang X., Fan Y., Li F. (2013). Preparation and properties of dynamically cured polypropylene (PP)/maleic anhydride–grafted polypropylene (MAH-g-PP)/calcium carbonate (CaCO_3_)/epoxy composites. J. Thermoplast. Compos. Mater..

[27] Lou X., Zhu C., Pan H., Ma J., Zhu S., Zhang D., Jiang X. (2016). Cost-Effective Three-Dimensional Graphene/Ag Aerogel Composite for High-Performance Sensing. Electrochim. Acta.

[28] Jiang X., Fan Y. (2012). Effect of the compatibilizer on the morphology and properties of dynamically cured PP/POE/epoxy blends. J. Appl. Polym. Sci..

[29] Sun Y., Fang Z., Wang C., Zhou A., Duan H. (2015). Incorporating nanoporous polyaniline into layer-by-layer ionic liquid-carbon nanotube-graphene paper: Towards freestanding flexible electrodes with improved supercapacitive performance. Nanotechnology.

[30] Berardi U., Iannace G. (2017). Predicting the sound absorption of natural materials: Best-fit inverse laws for the acoustic impedance and the propagation constant. Appl. Acoust..

[31] Ning J.F., Zhao G.P. (2014). Sound absorption characteristics of multilayer porous metal materials backed with an air gap. J. Vib. Control.

[32] Zhu W., Nandikolla V., George B. (2015). Effect of bulk density on the acoustic performance of thermally bonded nonwovens. J. Eng. Fabrics Fibers.

